# Men's more frequent predisposing factors in infectious endocarditis facilitate improvement of outcomes by shortening of diagnostic delay

**DOI:** 10.3389/fcvm.2024.1517288

**Published:** 2025-02-12

**Authors:** S. Andreß, K. Reischmann, S. Markovic, F. Rohlmann, B. Hay, W. Rottbauer, D. Buckert, S. d'Almeida

**Affiliations:** ^1^ Department of Internal Medicine II, Ulm University Hospital, Ulm, Germany; ^2^ Institute of Epidemiology and Medical Biometry, Ulm University, Ulm, Germany

**Keywords:** sex, infectious endocarditis, diagnostics, Duke criteria, risk factors, outcomes, diagnostic delay

## Abstract

**Introduction:**

Despite growing evidence for sex-specific differences in cardiovascular disease, sex is poorly considered in the management of infectious endocarditis (IE).

**Purpose:**

This study aimed to assess sex-specific aspects in diagnosing IE.

**Methods:**

All consecutive patients admitted at Ulm University Heart Center with suspected IE between 2009 and 2019 were included. IE was diagnosed using the Duke criteria. Risk factors, clinical presentation and in-hospital outcomes along with the impact of diagnostic delay were compared between male and female patients.

**Results:**

IE was diagnosed in 96 of 118 men (81.4%) and 33 of 45 women (73.3%) (*p* = 0.121). Time to diagnosis was similar between the groups (*p* = 0.598). Regarding patient characteristics, men were younger (65.5 vs. 74.3 years, *p* = 0.006). Men exhibited a higher prevalence of predisposing cardiac conditions (*p* = 0.012) due to a higher frequency of a history of implantable cardioverter defibrillator implantation (*p* = 0.004), and were more likely to have poor dental status (*p* = 0.001), and coronary artery disease (*p* = 0.002). The incidence of the complications of heart failure with reduced ejection fraction (*p* = 0.007) and new-onset dialysis (*p* = 0.012) were higher, the time in the intensive care unit (*p* = 0.012) longer. Male sex was the only independent risk factor for in-hospital mortality [*p* = 0.036, HR 4.127 (95%-CI 1.096-15.538)]. Notably, only in the male cohort, a shorter time to diagnosis was associated with a lower mortality rate (*p* = 0.035, optimal cut-point 3.5 days). Men diagnosed within 3.5 days had a mortality rate of 13.5% compared to 31.8% for those diagnosed later (*p* = 0.028).

**Conclusion:**

Men with suspected IE are younger, have more predisposing factors and experience a more complicated course of disease, while benefiting from early diagnosis. Therefore, recognizing the heightened risk profile specific to men during diagnosis can help to address their poorer prognosis.

## Graphical Abstract

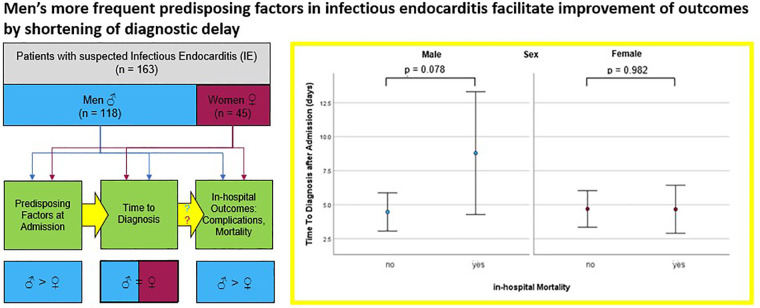

Summary of the study design and main findings.

## Introduction

Infective endocarditis (IE) is a life-threatening condition that poses several diagnostic and therapeutic challenges (1). It constitutes a major burden on the healthcare system with high morbidity and particularly elevated mortality rate of approximately 20% during the first hospitalization and 40% over five years (2). This burden is increasing over time, with the incidence of hospitalization for IE almost doubling between 2003 and 2016 (3). Notably, men are particularly affected (4, 5), with a 20% elevated incidence of IE (4) and a more pronounced increase in recent years (6). This development may be due to the differences in manifestation and progression of IE between men and women (7). Indeed, a higher proportion of culture-positive IE and Streptococcus viridans infection has been reported in men. While women are more prone to have infections of the left heart valves, aortic and mitral valve, men are more likely to develop vegetations of the tricuspid valve (5). Notwithstanding the substantial implications of these differences, sex is not among the criteria included in the Duke criteria, which are the global standard for the diagnosis of IE (8, 9). Additionally, demographic characteristics, risk factors and comorbidities, which influence the progression of the disease (10), differ between men and women (5). It has been documented that women with IE are more likely to have certain predisposing factors, including advanced age, prosthetic valve infection (10) and immunosuppression (5). Comorbidities associated with a poor prognosis are heterogeneously distributed between the sexes. Women are more likely to have chronic kidney disease (5), whereas men are more likely to have cardiovascular disease such as coronary artery disease (CAD) and heart failure (9–13). Moreover, quality of care appears to be inferior in women, as they tend to seek medical help later, are more likely to leave the hospital against medical advice (5, 10) and have lower rates of surgical intervention (5). Accordingly, women have been found to have higher mortality rates (5, 14). However, it is noteworthy, that the mortality is driven by the elderly (14). Another study only including patients aged 65 and older reported a higher mortality rate in men (6), thereby suggesting male sex to be a risk factor (6). Despite the prognostic relevance of these sex-based disparities, current guidelines for the diagnosis and management of IE do not account for sex (8, 9, 13). Overall, the data presented collectively illustrate that the role of sex in diagnosing IE remains poorly understood. Most importantly, they provide strong evidence that the implementation of sex-specific diagnostic management strategies could help to reduce the burden of IE (15). This study aimed to evaluate the sex-specific performance of the Duke criteria as standard for the diagnosis of IE and to identify possible sex-related differences, that can be incorporated to improve this algorithm.'

## Methods

### Study design

A monocentric, retrospective study was conducted, including all consecutive patients who presented with suspected IE at the Department of Medicine II at Ulm University Heart Center, Ulm, Germany, between December 2009 and November 2019. Patients who were hospitalized due to suspected IE were eligible for inclusion in the study. All patients who were suspected of having IE were included, regardless of whether a diagnosis of definitive IE was made at a later stage. All patients were subjected to recurrent assessment according to the Duke criteria as outlined by the European Society of Cardiology (ESC) in 2023 (9), within 24 h of admission and again throughout the duration of their initial hospitalization until discharge. The diagnosis provided at the time of discharge was considered the definitive diagnosis. All patients were treated in accordance with to the prevailing guidelines at that time (16, 17). Patients who were readmitted to the hospital for a second instance of suspected IE relapse were excluded from the study. Based on their sex, the patients were divided into the female and the male group. Sex was considered in accordance with the SAGER (Sex and Gender Equity in Research) Guidelines to ensure appropriate analysis and reporting of biological differences between male and female patients.

The study conforms to the guidelines of the Declaration of Helsinki, adheres to the STROBE statement and was approved by the local ethics committee (ethics application number University Ulm 90/20).

### Data collection, clinical assessment, imaging and laboratory procedures

Demographic, clinical, imaging and laboratory data at the time of admission and during the hospital stay were extracted from our patient management system by two physicians and adjudicated by a third physician in case of any kind of disagreement. The positivity of the Duke criteria was assessed to evaluate the presence of IE resulting in the diagnosis of definitive IE, or no confirmation of the suspicion in case of possible or rejected IE (9, 17). For this purpose, clinical assessment and targeted cardiovascular examinations such as transthoracic echocardiography and, if appropriate, transesophageal echocardiography and Positron Emission Tomography-Computed Tomography (PET-CT) were performed according to the recommendations. In addition, 12-lead electrocardiography (ECG) and laboratory blood tests were performed. Left ventricular systolic function (left ventricular ejection fraction, LVEF) was measured either by echocardiography (EPIQ 7, Koninklijke Philips N.V., Eindhoven, The Netherlands) or cardiac ventriculography during cardiac catheterization and categorized as normal, mildly impaired, moderately impaired, or severely impaired, according to guideline-specific recommendations (18). Blood samples were taken for measurement of highly sensitive cardiac troponin T (hsTnT), N-Terminal pro-B-Type Natriuretic Peptide (NT-proBNP), creatinine and estimated glomerular filtration rate (eGFR), c-reactive protein (CRP), procalcitonin (PCT), leukocytes and neutrophils. Blood cultures and urine samples were collected for microbiological and virological pathogen testing and gathered retrospectively. Definitive IE was categorised into native valve, prosthetic valve and lead or device-related infections according to the cardiac structures involved. All data were collected as part of the clinical routine.

### Endpoints

Endpoints were differences between male and female patients regarding diagnosis, baseline characteristics, disease course and outcomes, with a focus on the feasibility and impact of reducing diagnostic delay. First, demographic factors, comorbidities, predisposing factors and symptoms of IE were assessed and compared. In particular, the frequency of positive Duke criteria and diagnosis of IE were assessed, if appropriate recurrently, along with the time to diagnosis. Duke criteria were typical imaging and microbiological findings, fever > 38°C, the presence of a predisposing heart disease such as prosthetic valve, bicuspid aortic valve, congenital cardiac malformation, intracardial device such as implantable cardioverter-defibrillator (ICD) or pacemaker (PM), structural cardiomyopathy or intravenous drug abuse, vascular phenomena and immunological phenomena, according to the guidelines (9). Other predisposing factors assessed included poor dental status and immunosuppression. Secondary, the in-hospital course of disease and outcomes were assessed and compared between the groups. These included complications and hospitalization data. Complications included cardiac failure, acute kidney injury requiring new-onset of dialysis, and mortality. Length of total and intensive care unit (ICU)-stay and number of ICU-stays were assessed. Finally, the effect of delayed diagnosis on mortality was examined. For this purpose, the association of the time between admission and diagnosis and mortality was assessed. Time of diagnosis was defined as the point of time at which the final diagnosis in terms of IE was made, irrespective of the result. Where appropriate, the critical time period and its effect on mortality were assessed. In this term, the time to diagnosis was compared between the subgroups with and without mortality. The observation period was the time of the interventional hospital stay until discharge.

### Statistical analysis

Continuous variables were presented as mean ± standard deviation or median together with the interquartile range (IQR) as appropriate. The Kolmogorov-Smirnov test was used to assess the normal distribution of continuous parameters. If a metric variable was not normally distributed at any measurement date, all values were presented as median together with the IQR. Categorial variables were described as numbers and percentages. Student's *t*-test, Mann-Whitney *U*-Test or chi-square test was used to compare variables between groups where appropriate. Multinominal cox regression analysis was used to calculate age-adjusted mortality. In addition, multivariate cox regression analysis was performed to assess the association between male sex and the risk of in-hospital mortality while adjusting for potentially confounding baseline characteristics. Sex was included as the primary independent variable. Baseline characteristics with a *p*-value < 0.05 in the univariate comparisons between the groups were included in the multivariate analysis. The enter method was applied for variable inclusion in the multivariate cox regression analysis, and the proportional hazards assumption was tested by creating interaction terms, with violations addressed by stratification or exclusion of problematic variables. The strength of the association was expressed as hazard ratio (HR) with 95%-confidence interval (CI). Binary regression, and additionally Pearson correlation analysis, were used to assess the effect of time to diagnosis on mortality and were performed separately for men and women. The performance of the model was assessed by receiver operating characteristic (ROC) analysis estimating sensitivity, specificity, and area under the curve (AUC). The optimal cut-off was calculated using the Youden index.

Parameters with a *p-*value < 0.05 were considered statistically significant. Statistical analysis was performed using SPSS Statistics 29 software (Version 2022, IBM, Armonk, NY, USA). Due to the exploratory nature of this study, all results of statistical tests have to be interpreted as generating hypotheses.

## Results

### Study population

**Figure 1:**
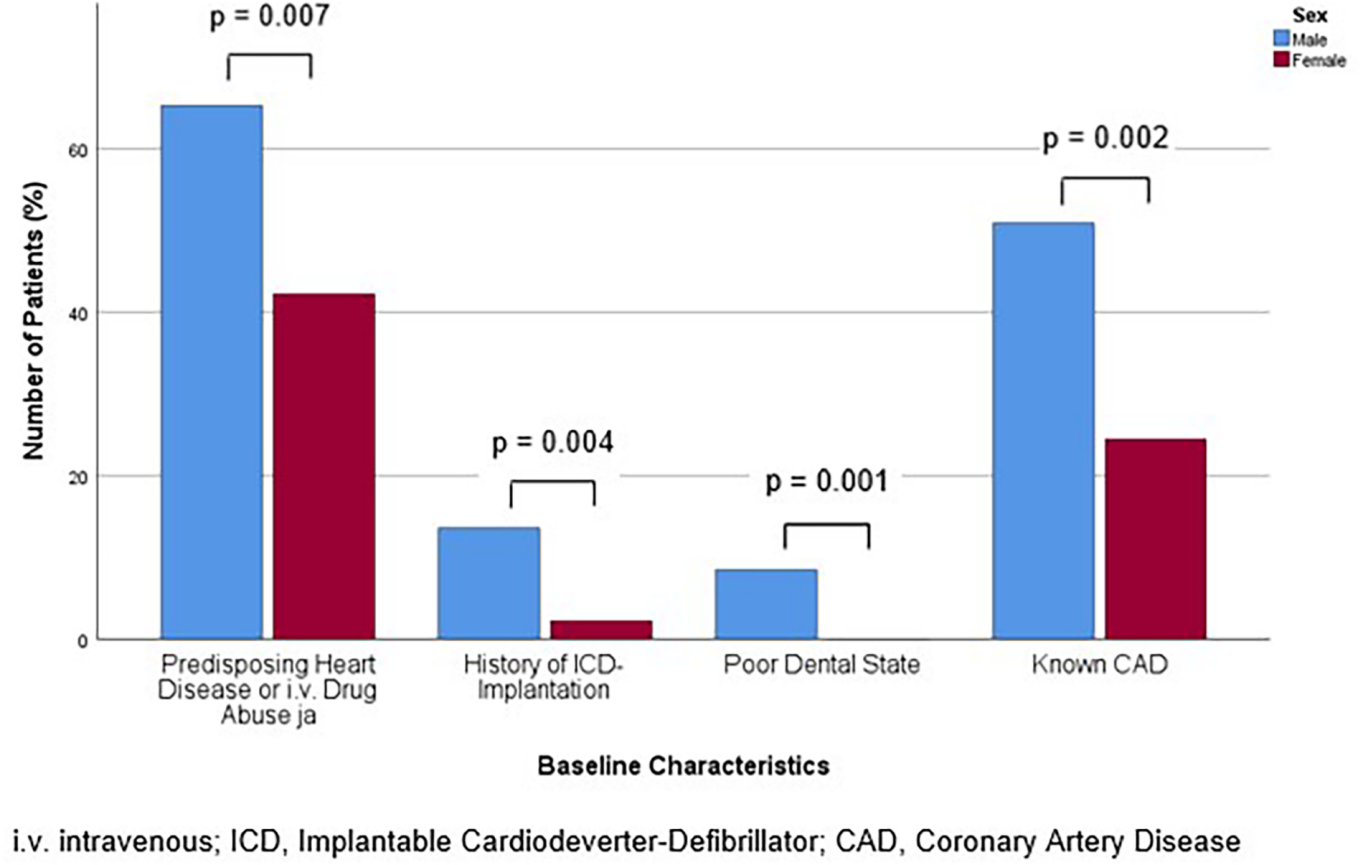
Bar chart for the percentage of patients with positive Duke criteria corresponding to the diagnosis of Infectious Endocarditis (IE) according to the Duke criteria overall, at admission and during the hospital stay, for males and females, compared with Mann-Whitney-U- or Students' *t*-test, as appropriate.

A total of 163 patients received inpatient treatment at our tertiary care center for suspected IE between December 2009 and November 2019. Of the total number of patients, 118 (72.4%) were men (male group) and 45 (27.6%) were female (female group). The baseline data for both groups are presented in Table 1. The median age was significantly lower in the male group compared to the female group [65.5 (56.3, 75.4) years vs. 74.3 (70.2, 82.7) years (*p* = 0.006)]. The male group exhibited a higher prevalence of CAD (50.8% vs. 24.4%, *p* = 0.002) (Figure 1) and a history of myocardial infarction (22.0% vs. 2.2%, *p* < 0.001). In the female group, the prevalence of thyroid dysfunction was higher (35.6% vs. 13.6%, *p* = 0.007), attributable to a greater proportion of women with hypothyroidism (31.1% vs. 11.9%, *p* = 0.014). The characteristics of IE are also shown in Table 1. In the male cohort, IE was more frequently observed in the tricuspid valve (9.3% vs. 2.2%, *p* = 0.044). Otherwise, there were no significant differences in the patients' baseline characteristics. Notably, the type of endocarditis, including native valve and prosthetic valve infections, as well as the proportion with lead infection, were comparable. Furthermore, there was no significant difference in the proportion of patients in whom vegetations or bacteria were detected. In both groups, bacteriemia was primarily attributable to Staphylococcus aureus, which was identified in approximately 30% of the patients (male group: 33.1% vs. female group: 28.9%, *p* = 0.613). Subsequently, infection with Enterococcus faecalis/faecium (male group 12.7% vs. female group 11.1%, *p* = 0.921) and Streptococcus viridans (male group 24.6% vs. female group 4.4%, *p* = 0.124) was observed. The proportion of men with infection with Streptococcus gallolyticus (bovis) was low (1.7% vs. female group 0.0%, *p* = 0.383). The HACEK group of bacteria (comprising Haemophilus aphrophilus and haemophilus, Aggregatobacter actinomycetemcomitans, Cardiobacterium hominis, Eikenella corrodens and Kingella kingae) were not identified in the present cohort.

**Table 1:** Baseline characteristics of males and females.

	Male-group	Female-group	*p*-value
	*n* = 118	*n* = 45	
Patients characteristics			
Age (years)	65.5 (56.3, 75.4)	74.3 (70.2, 82.7)	**0**.**006**
BMI (kg/m^2^)	27.29 ± 5.06	26.83 ± 8.45	0.762
Known CAD (%)	60/118 (50.8)	11/45 (24.4)	**0**.**002**
History of MI (%)	26/118 (22.0)	1/45 (2.2)	**<0.001**
Rhythmological Disorder (%)	62/118 (52.5)	26/45 (57.8)	0.552
Arterial hypertension (%)	76/118 (64.4)	31/45 (68.9)	0.593
Diabetes mellitus (%)	36/118 (30.5)	9/45 (20.0)	0.158
Type I (%)	2/118 (1.7)	0/45 (0)	0.383
Type II (%)	34/118 (28.8)	9/45 (20.0)	0.233
CKD (%)	37/118 (31.4)	14/45 (31.1)	0.976
CKD Stage (%)			0.481
CKD 0 (%)	79/118 (66.9)	31/45 (68.9)	
CKD I (%)	19/118 (16.1)	8/45 (17.8)	
CKD II (%)	1/118 (0.8)	1/45 (2.2)	
CKD III (%)	13/118 (11.0)	4/45 (8.9)	
CKD IV (%)	4/118 (3.4)	1/45 (2.2)	
CKD V (%)	2/118 (1.7)	0/45 (0)	
Preexisting Dialysis (%)	5/118 (4.2)	2/45 (4.4)	0.955
Cancer (%)	11/118 (9.3)	4/45 (8.9)	0.932
Immunosuppression (%)	8/118 (6.8)	3/45 (6.7)	0.980
Thyroid dysfunction (%)	16/118 (13.6)	16/45 (35.6)	**0**.**007**
Type Hypothyreosis (%)	14/118 (11.9)	14/45 (31.1)	**0**.**014**
Type Hyperthyreosis (%)	2/118 (1.7)	2/45 (4.4)	0.412
Rheumatic Disease (%)	19/118 (7.6)	3/45 (6.7)	0.835
Characteristics of IE			
Type of Endocarditis			0.066
NVE (%)	63/93 (67.7)	29/35 (82.9)	0.122
PVE (%)	30/93 (32.3)	6/35 (17.1)	0.122
Vegetations (%)	101/118 (85.6)	38/45 (84.4)	0.854
Location			
AV (%)	52/118 (44.1)	16/45 (35.6)	0.323
MV (%)	43/118 (36.4)	24/45 (53.3)	0.050
TV (%)	11/118 (9.3)	1/45 (2.2)	**0**.**044**
PV (%)	1/118 (0.8)	0/45 (0)	0.539
Leads (%)	16/118 (13.6)	5/45 (11.1)	0.679
Microbiological finding (%)	89/118 (75.4)	32/45 (71.1)	0.576
Staphyolococcus aureus (%)	39/118 (33.1)	13/45 (28.9)	0.613
Streptococcus viridans (%)	29/118 (24.6)	2/45 (4.4)	0.124
Streptococcus gallolyticus (bovis) (%)	2/118 (1.7)	0/45 (0)	0.383
HACEK (%)	0/118 (0)	0/45 (0)	1.000
Enterococcus faecalis/faecium (%)	15/118 (12.7)	5/45 (11.1)	0.921
CRP at Admission (mg/dl)	107.4 ± 98.4	93.1 ± 82.1	0.394
PCT at Admission (µg/L)	4.9 ± 8.0	4.5 ± 6.1	0.948
Leukocytes at Admission (Giga/L)	11.8 ± 8.0	12.08 ± 6.6	0.836
Neutrophilia at Admission (%)	9/17 (52.9)	8/11 (72.7)	0.313

Significant differences are presented in bold.

BMI, body mass index; CAD, coronary artery disease; MI, myocardial infarction; CKD, chrnic kidney disease; NVE, native valve endocarditis; PVE, prosthetic valve endocarditis; AV, aortic valve; MV, mitral valve; TV, tricuspid valve; PV, pulmonal valve; HACEK, haemophilus aprophilus and paraprophilus, aggregatobacter actinomycetemcomitans, cardiobacterium hominis, eikanella corrodens, kingella kingae; CRP, C-reactive protein; PCT, procalcitonine.

### Diagnosis, symptoms and risk factors of IE

To evaluate the representation of any sex-specific differences in the diagnostic guidelines, a comparison was conducted between the presence of these criteria among males and females. In addition, the presence of other known risk factors and symptoms of IE were compared to assess their sex-specific expression.

**Figure 2:**
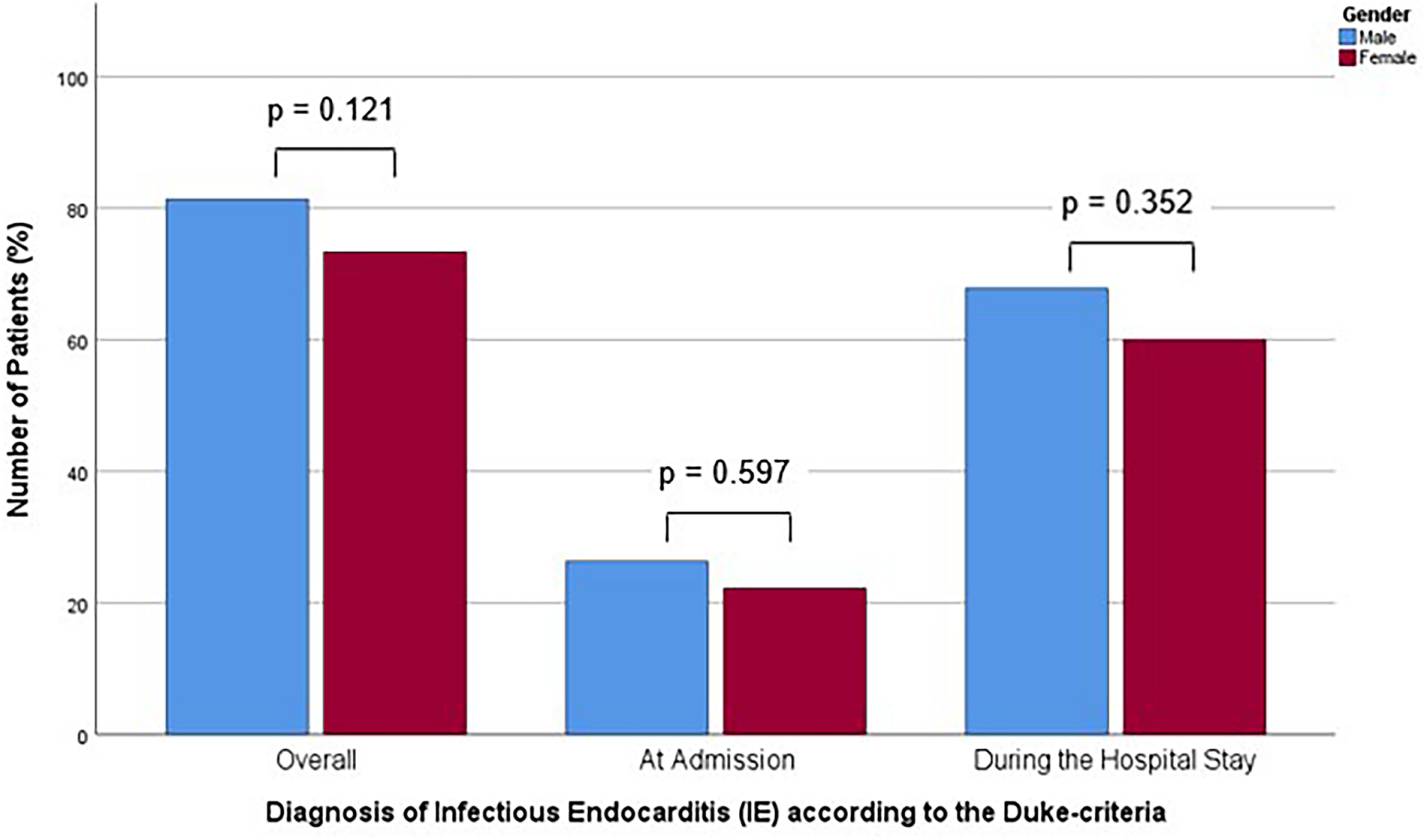
Bar chart for the percentage of patients with baseline characteristics with significant difference in prevalence between the sexes for males and females, compared with Mann-Whitney-U- or Students' *t*-test, as appropriate.

A total of 129 of 163 patients (79.1%) were diagnosed with definitive IE until discharge. This affected 96 of 118 males (81.4%) and 33 of 45 females (73.3%), representing similar proportions of both groups (*p* = 0.121). At the time of admission, 31 (26.3%) males and 10 (22.2%) females were diagnosed with IE according to the Duke criteria. This was not a statistically significant difference between the sexes (*p* = 0.597). During the hospital stay, out of all 163 patients, 80 patients (67.8%) in the male group and 27 (60.0%) in the female group, were found to have positive Duke criteria, corresponding to the diagnosis of IE, which was also not a statistically significant difference (*p* = 0.352). The diagnostic delay, as determined by the time-to-diagnosis following admission (*p* = 0.598), was comparable between the two groups (Table 2). The proportion of patients with positive Duke criteria, corresponding to the diagnosis of IE, for the different time points assessed for both sexes is shown in Figure 2.

**Table 2:** Diagnosis of IE according to the Duke criteria for males and females.

	Male-group	Female-group	*p*-value
	*n* = 118	*n* = 45	
Overall (%)	96/118 (81.4)	33/45 (73.3)	0.121
At Admission (%)	31/118 (26.3)	10/45 (22.2)	0.597
During the Hospital Stay (%)	80/118 (67.8)	27/45 (60.0)	0.352
Time from Admission to Diagnosis (days)	5.4 ± 8.0	4.7 ± 4.0	0.598
Number with positive Duke major Criteria at Admission			0.850
0 (%)	50/118 (42.4)	17/45 (37.8)	
1 (%)	42/118 (35.6)	19/45 (42.2)	
2 (%)	26/118 (22.0)	9/45 (20.0)	
Number with positive Duke minor Criteria at Admission			**0** **.** **024**
0 (%)	14/118 (11.9)	12/45 (26.7)	
1 (%)	43/118 (36.4)	16/45 (35.6)	
2 (%)	46/118 (39.0)	14/45 (31.1)	
3 (%)	14/118 (11.9)	3/45 (6.7)	
4 (%)	1/118 (0.8)	0/45 (0)	

Significant differences are presented in bold.

In line with the aforementioned results, at admission, the frequency of positivity for the major criteria, which are positive imaging or microbiological detection of bacteria typical for IE, was similar (*p* = 0.850). However, men exhibited a higher frequency of positive minor criteria, including fever, predisposing heart disease or intravenous drug abuse, vascular phenomena, immunologic phenomena, microbiological or imaging criteria suspicious for IE, but not fulfilling the corresponding major criterion (*p* = 0.024). An analysis of the individual criteria revealed that predisposing heart disease or intravenous drug abuse was more prevalent in males (65.3% vs. 42.2%, *p* = 0.007). This was primarily due to the high incidence of predisposing heart disease (61.9% vs. 40.0%, *p* = 0.012). In detail, a history of ICD implantation affected a greater proportion of males (13.6% vs. 2.2%, *p* = 0.004). Regarding the other risk factors, poor dental status was asserted exclusively and more frequently in males (8.5% vs. 0%, *p* = 0.001). Additionally, men were more likely to present with splenomegaly (33.1% vs. 13.3%, *p* = 0.004) (Table 3). The prevalence of the predisposing baseline characteristics of significant difference for men and women are displayed in Figure 1.

**Table 3:** Frequency of the Duke criteria, risk factors and clinical symptoms of IE for males and females.

	Male-group	Female-group	*p*-value
	*n* = 118	*n* = 45	
Duke major criteria			
Imaging positive for Endocarditis (%)	43/118 (36.4)	22/45 (48.9)	0.149
Microbiological major Criterium (%)	51/118 (43.2)	15/45 (33.3)	0.246
Duke minor criteria			
Fever > 38°C (%)	58/118 (49.2)	21/45 (46.7)	0.778
Predisposing Heart Disease or intravenous Drug Abuse (%)	77/118 (65.3)	19/45 (42.2)	**0**.**007**
Predisposing Heart Disease (%)	73/118 (61.9)	18/45 (40.0)	**0**.**012**
Heart Valve Prothesis (%)	37/118 (31.4)	9/45 (20.0)	0.128
CIED (ICD/PM/CRT) (%)	30/118 (25.4)	9/45 (20.0)	0.471
ICD (%)	7/118 (13.6)	1/45 (2.2)	**0**.**004**
PM (%)	26/118 (22.0)	8/45 (17.8)	0.553
Structural Heart Disease (%)	10/118 (8.5)	2/45 (4.4)	0.382
Intravenous Drug Abuse (%)	6/118 (5.1)	1/45 (2.2)	0.423
Vascular Phenomena (%)	34/118 (28.8)	9/45 (20.0)	0.233
Cerebral Emboly (%)	22/118 (18.6)	6/45 (13.3)	0.425
Arterial Embolisation (%)	12/118 (10.2)	3/45 (6.7)	0.502
Cerebral Hemorrhagic (%)	6/118 (5.1)	1/45 (2.2)	0.423
Septic Lung Infarct (%)	2/118 (1.7)	0/45 (0)	0.383
Mycotic Aneurysm (%)	1/118 (0.8)	0/45 (0)	0.539
Janeway Lesion (%)	9/118 (7.6)	2/45 (4.4)	0.472
Immunologic Phenomena (%)	4/118 (3.4)	2/45 (4.4)	0.701
Glomerulonephritis (%)	3/118 (2.5)	1/45 (2.2)	0.907
Osler-Knots (%)	1/118 (0.8)	0/45 (0)	0.539
Roth-Spots (%)	0/118 (0)	0/45 (0)	1.000
Positive Rheuma factor (%)	0/118 (0)	0/45 (0)	1.000
Microbiological minor Criterion (%)	8/118 (6.8)	3/45 (6.7)	0.980
Risk factors			
Poor Dental State (%)	10/118 (8.5)	0/45 (0)	**0**.**001**
Clinical symptoms			
Night sweats/Chills (%)	34/118 (28.8)	11/45 (24.4)	0.580
Weight loss (%)	31/118 (26.3)	9/45 (20.0)	0.409
Splenomegaly (%)	39/118 (33.1)	6/45 (13.3)	**0**.**004**
Spleen Infarction (%)	9/118 (7.5)	3/45 (6.7)	0.835
Hepatomegaly (%)	24/118 (20.3)	7/45 (15.6)	0.490
New Cardiac Murmur (%)	19/118 (16.1)	8/45 (17.8)	0.789

Significant differences are presented in bold.

CIED, cardiac implantable electronic device; ICD, implantable cardioverter-defibrillator; PM, pacemaker; CRT, cardiac resynchronization therapy.

### Course of disease

In order to assess the impact of sex-specific factors on the course of disease, the complications and outcomes of males and females were compared.

**Figure 3:**
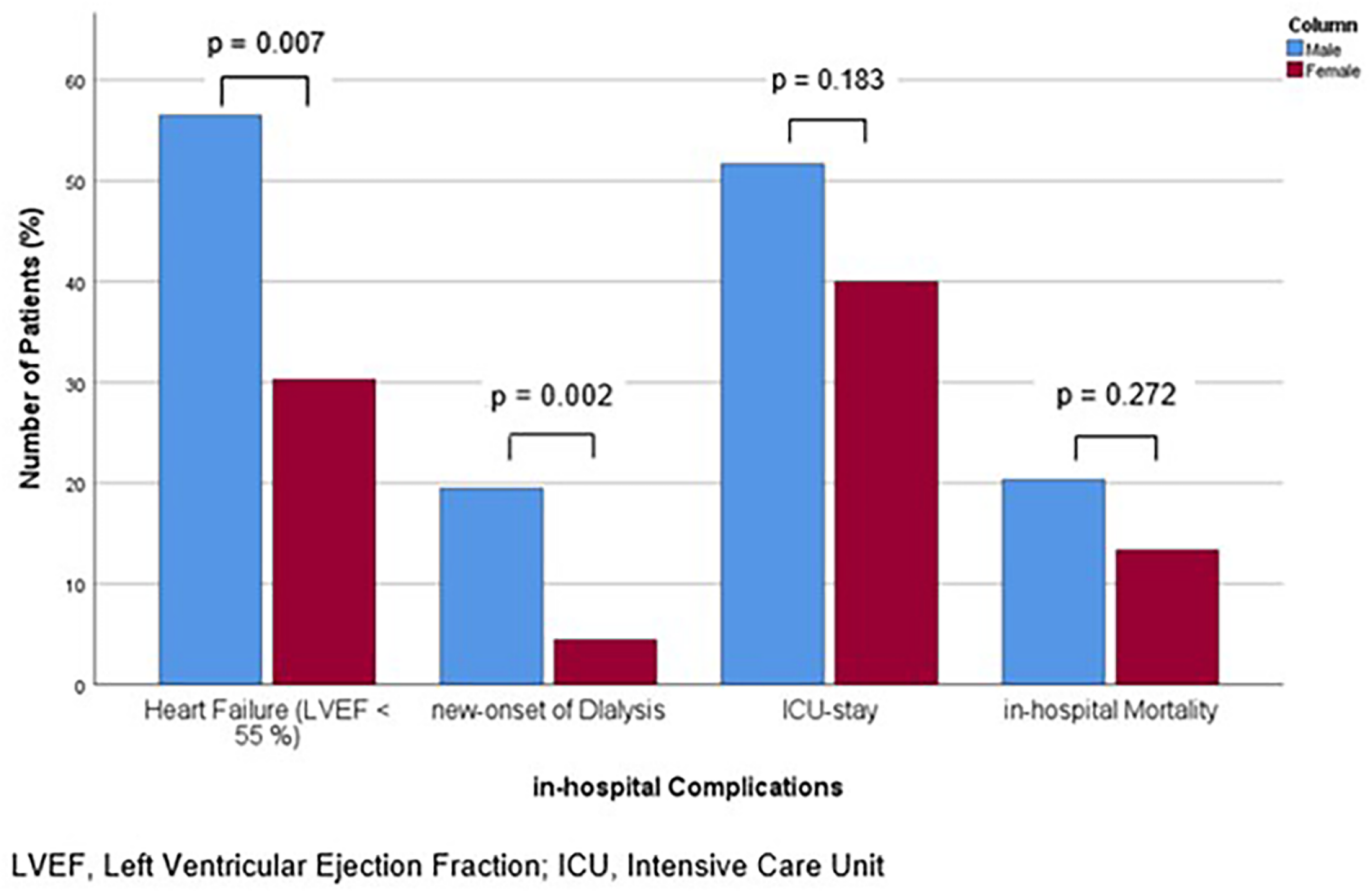
Bar chart for the percentage of patients with in-hospital complications of heart failure with reduced ejection fraction, new-onset of dialysis, ICU-stay and in-hospital mortality, for males and females, compared with Mann-Whitney-U- or Students' *t*-test, as appropriate.

A reduced cardiac function was observed more frequently in men (56.5% of male patients vs. 30.3% of female patients, *p* = 0.007) and their grade of LVEF was lower (*p* = 0.017). Furthermore, male patients exhibited elevated creatinine levels, both at admission [116.5 (83.8, 178.0) vs. 93.0 (70.0, 157.0) µmol/L, *p* = 0.008] and throughout the course of their hospitalization [peak 172.5 (103.5, 312.8) vs. 109.0 (88.0, 198.5) mmol/L, *p* = 0.006]. Consistently, the incidence of new-onset dialysis was higher in the male cohort (19.5% vs. 4.4%, *p* = 0.002). With regard to the treatment measures, the time until the initial administration of antibiotics was comparable (*p* = 0.276). Also, the overall length of hospitalization was comparable between the sexes (*p* = 0.065), but men spent a significantly longer time in the ICU [12.0 (6.0, 19.0) vs. 6.0 (3.0, 11.0), *p* = 0.012]. The proportion of patients, who required ICU-admission did not differ between the sexes (*p* = 0.183). Notably, the absolute mortality rate was comparable between the sexes (*p* = 0.272), while age-adjusted mortality was significantly higher in males (*p* = 0.013, HR 3.631, 95%-CI 1.308–10.075) (Table 4). The proportions of patients affected by the aforementioned complications for both sexes are shown in Figure 3. Multivariate analysis including all baseline characteristics of significant difference in the univariate comparisons between the groups (age, known CAD, history of myocardial infarction, predisposing heart disease or intravenous drug abuse, ICD, poor dental state, thyroid dysfunction, splenomegaly, location TV, hypothyroidism, predisposing heart disease) revealed male sex as (the only) independent risk factor for in-hospital mortality (*p* = 0.036, HR 4.127 (95%-CI 1.096–15.538). The covariates included are shown in Supplementary Table S1.

**Table 4:** Course of disease and outcomes of males and females.

	Male-group	Female-group	*p*-value
	*n* = 118	*n* = 45	
Complications of organ failure			
LVEF grade			**0**.**017**
Normal (>= 55%) (%)	47/108 (43.5)	23/33 (69.7)	
Mildly impaired (45%–54%) (%)	27/108 (25.0)	4/33 (12.1)	
Moderately impaired (35%–44%) (%)	12/108 (11.1)	3/33 (9.1)	
Severely impaired (<35%) (%)	22/108 (20.4)	3/33 (9.1)	
Reduced (%)	61/108 (56.5)	10/33 (30.3)	**0**.**007**
Troponin T (ng/L)	219.0 ± 556.4	87.1 ± 108.3	0.274
NT-proBNP (pg/ml)	9,367.8 ± 12,021.6	10,104.6 ± 11,113.9	0.824
Initial Creatinine initial (µmol/L)	116.5 (83.8, 178.0)	93.0 (70.0, 157.0)	**0**.**008**
Peak Creatinine (µmol/L)	172.5 (103.5, 312.8)	109.0 (88.0, 198.5)	**0**.**006**
New-onset of Dialysis (%)	23/118 (19.5)	2/45 (4.4)	**0**.**002**
Treatment measures			
Time until first Application of Antibiotics (days)	2.4 ± 6.4	1.3 ± 3.0	0.276
Length of Antibiotics (days)	40.2 ± 23.0	36.6 ± 17.6	0.369
Surgery (%)	52/118 (54.1)	13/45 (28.9)	0.069
Interventional Renovation (%)	1/118 (0.8)	0/45 (0)	0.539
Device Revision (%)	7/118 (5.9)	2/45 (4.4)	0.712
Hospitalization and outcomes			
Length of Hospital Stay (days)	32.0 ± 23.3	25.0 ± 16.1	0.065
ICU stay (%)	61/118 (51.7)	18/45 (40.0)	0.183
Length of ICU-stay (days)	12.0 (6.0, 19.0)	6.0 (3.0, 11.0)	**0**.**012**
Number of ICU-stays average	1.3 ± 0.6	1.3 ± 0.5	0.944
1 (%)	44/118 (37.3)	12/45 (26.7)	
2 (%)	13/118 (11.0)	6/45 (13.3)	
3 (%)	4/118 (3.4)	0/45 (0)	
In-hospital Mortality (%)	24/118 (20.3)	6/45 (13.3)	0.272
Age-adjusted			**0** **.** **013**
			HR 3.631, 95%-CI 1.308–10.075
Adjusted for all baseline characteristics of significant difference in the univariate comparisons between the groups (*p* value < 0.05)			**0** **.** **036**
			HR 4.127, 95%-CI 1.096–15.538

Significant differences are presented in bold.

LVEF, left ventricular ejection fraction; ICU, intensive care unit; HR; hazard ratio; CI, confidence interval.

### Complication rates of patients with and without confirmation of IE

To assess the comparability of patients who were definitively diagnosed with IE and who were not, we calculated and compared the complication rates of patients who were definitively diagnosed with IE and those who were not. Here, no differences were observed in the complication rates of new-onset of dialysis (15.5% vs. 14.7%, *p* = 0.909), heart failure 43.4% vs. 41.2%, (*p* = 0.967), length of ICU-stay [10.00 (5.00, 16.00) days vs. 15.00 (3.25, 19.00) days, *p* = 0.465] and in-hospital mortality (17.1% vs. 23.5%, *p* = 0.389) between the two groups.

### Effect of diagnostic delay

In order to evaluate the potential benefits of reducing the time taken to reach a diagnosis, we examined the effect of diagnostic delay on mortality rates.

**Figure 4:**
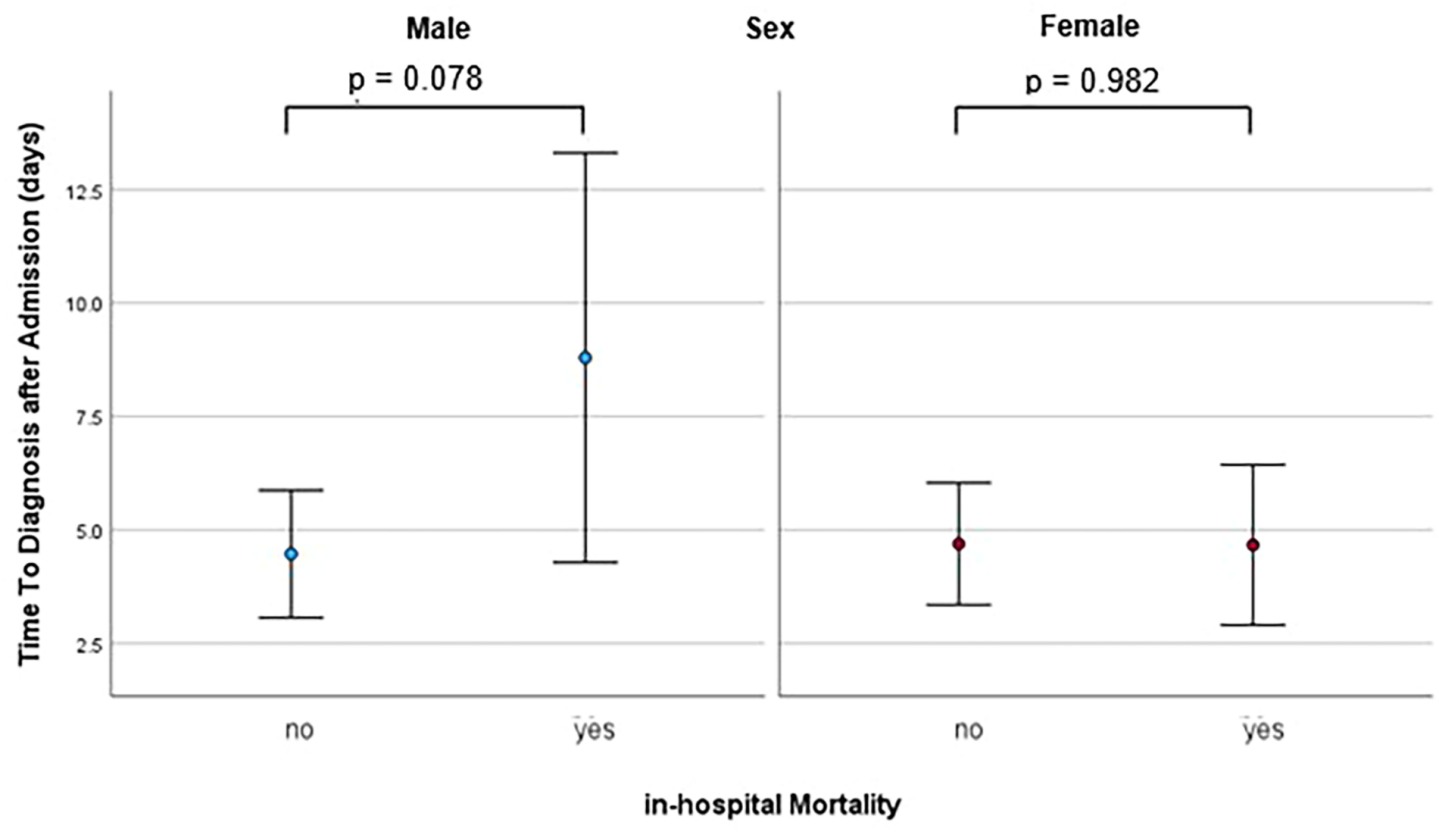
Dot plot with error bars showing the mean and standard deviation of time to diagnosis in patients with and without in-hospital mortality, seperately for males and females, calculated with binary regression analysis.

Deaths during the initial hospital stay occurred in 24 out of 118 patients (20.3%) in the male group and 6 out of 45 patients (13.3%) in the female group. In the male group, both binary regression analysis and Pearson correlation analysis revealed a statistically significant association between time to diagnosis and mortality (*p* = 0.035 and 0.017, respectively). ROC analysis for the prediction of mortality by time to diagnosis yielded an AUC of 0.627 (95%-CI 0.489–0.765, *p* = 0.078)**.** The optimal cut-off point, as calculated by the Youden index, was 3.5 days, exhibiting a sensitivity of 58.3% and a specificity of 68.1%. Male patients diagnosed within this timeframe exhibited an event rate of 13.5% (10 of 74 patients), in comparison to 31.8% for those with a longer time to diagnosis (14 of 44 patients) (*p* = 0.028). Accordingly, there was a strong trend towards a longer time to diagnosis in patients who died compared to those who survived (8.8 ± 11.1 vs. 4.5 ± 6.8 days, *p* = 0.071). In contrast, no significant association between time to diagnosis and mortality was observed among women (*p* = 0.988 for both analyses). Furthermore, there time-to-diagnosis was comparable between female patients with and without mortality (*p* = 0.982). The time to diagnosis for patients with and without mortality, presented separately for each sex, is illustrated in Figure 4.

## Discussion

The present study analyzed the differences between men and women in IE in terms of risk factors and symptoms at baseline, outcomes, and the impact of diagnostic delay. Although the frequency of diagnosis in accordance with the current guidelines was comparable between the sexes, the prevalence of predisposing conditions was higher in men. In detail, predisposing heart disease, specifically a history of ICD-implantation, as well as poor dental state and known CAD were more prevalent in men. Furthermore, the course of disease in men was more severe in terms of organ failure. Importantly, males sex was the only independent risk factor for in-hospital mortality. Notably, a shorter time to diagnosis was found to be associated with lower in-hospital mortality only in male patients. The study design and the main findings are illustrated in the graphical abstract.

### Purpose: meeting the shortcomings of the Duke criteria by adding new criteria

Diagnosing IE remains challenging nowadays and a definitive diagnosis is often elusive. Despite the transition from the old Israel Beth criteria to the Duke Criteria and subsequent updates, the latest iteration still exhibits a sensitivity of only 84.2%, while its specificity is better with 93.9% (19). This discrepancy is also reflected in the guidelines that differentiate between definitive IE (DIE), possible IE (PIE), and rejected IE (RIE) (10). In light of these data, indicating that IE is underdiagnosed, all patients diagnosed with suspicion and thus a certain probability of having IE were included in the present study to investigate potential new criteria. The high specificity of the Duke criteria of 93.9% (19) indicates, that this inclusion criterion of “suspected IE” ensures the capture of an accordingly high percentage of patients with IE. Our results of similar outcomes of patients with and without confirmation of the suspicion of IE reinforce this hypothesis that failure to definitively diagnose IE is not solely due to the absence of IE, but rather to the lack of diagnostic sensitivity of the Duke criteria. These results underscore the suitability of the present cohort of patients with “suspected IE” for investigating additional criteria to enhance the diagnostic performance of the Duke criteria, particularly in terms of sensitivity.

### Sex-specific performance of the Duke criteria in diagnosing IE

Diagnosis of IE is challenging due to its heterogeneous presentation, but decisive for treatment and thus prognosis (1). The Duke criteria represent the global standard for diagnosing IE as evidenced by their acceptance by the major cardiac societies of the ESC (8, 9) and the American College of Cardiology (ACC) (20) and its widespread utilization (21). Since there is compelling evidence that IE manifests differently between males and females (7), but sex is not considered in the Duke score as a criterion (9), we assessed and compared the sex-specific performance of these guidelines. In our study, out of the in total approximately 80% of patients who were diagnosed with IE using the Duke score, only approximately one quarter received this diagnosis even at the time of admission. Notably, while the number of positive minor criteria at admission was higher in men, this was not reflected in the overall positivity of the score. Conversely, the proportion of patients diagnosed with IE was comparable between men and women, indicating that sex does not significantly impact diagnosis by the Duke score. Furthermore, the time to positivity of the score was comparable, which serves to reinforce the absence of sex-based disparities. Thus, our results show a reflection of sex-specific aspects by the Duke criteria, but indicate that these disparities are underrepresented in the resulting score. In line with this, the suitability of the Duke criteria is demonstrated by the consistence of the core criteria throughout various stages of development, updates and validations (8, 9, 20, 22–24) and the high performance with a sensitivity of 84.2% and a specificity of 93.9% of the most recent iteration (19). On the other hand, in consistence with the shortcomings of the score found in our study, the WikiGuidelines Group Consensus Statement already pointed out limitations due to the absence of high-quality studies that definitely determine the most appropriate diagnostic schema (21). Thus, our findings underline the high performance of the Duke criteria, but emphasize the recently indicated limitations, as the implied elevated risk in men is not reflected in the total score and, about three-quarters of patients had a delayed diagnosis.

### Men more frequently have predisposing baseline characteristics

Given this higher frequency of minor criteria in men, it is essential to identify the underlying causes to enable appropriate assessment and care of IE. In detail, we observed a higher prevalence of a cardiac predisposition, affecting nearly two-thirds of the male patients, which was due to a higher frequency of patients with a history of ICD implantation. Additionally, a higher proportion of men exhibited poor dental status, with approximately 10% presenting with this condition. Furthermore, men were more likely to have the comorbidity CAD, which affected approximately half of them, and a history of myocardial infarction. These findings of the present study consistently indicate that men are at a higher risk of IE, as the association of these conditions with IE is well documented. Recent studies have reaffirmed congenital heart disease, a history of valve replacement, repair, degenerative valve disease, previous IE, a history of invasive procedures in the previous 60 days, intravenous drug use, and chronic intravenous access as risk factors (25–29). Congenital heart disease and intravenous illicit drug use have even been reported to be the most common predisposing conditions (21) and are already included in the Duke score as a minor criterion (9). In detail, approximately 10% of all IE cases have been found to be associated with drug use (30, 31). The implications of cardiac predisposition is demonstrated by the recommendation of the 2007 update of the American Heart Association, which suggests that IE prophylaxis for dental procedures should only be given to affected patients (32). Intravenous drug abuse has even been shown to be a complicating factor, as it is associated with high recurrence (33). The presence of a foreign intracardiac device, such as an ICD, is an obvious predisposing factor to IE, as it provides a potential portal of entry and medium for bacterial growth (9). The implication of this condition (9) is outlined by the high mortality rate associated with device-related IE of 10%–30% (34). The risk of developing IE associated with poor dental status is obvious, as this facilitates the spread of bacteria and their migration to the heart. This predisposition is even reinforced by the guidelines, which consider this condition for risk stratification in IE and as a reason for application of antibiotic prophylaxis (9). Accordingly, a recent study even identified poor oral hygiene as the primary factor contributing to the development of oral streptococcal IE (35). Although the in males more common comorbidity CAD with history of myocardial infarction not directly contributes to the risk factors mentioned in the guidelines (9), it may be a predisposing factor. Its effects, ischemia and myocardial damage with resulting structural cardiomyopathy, are among the predisposing cardiac conditions that form part of the Duke score (9). In addition, CAD may have an indirect negative impact on prognosis through its association with myocardial infarction and reduced LVEF, both of which are part of the Charlton comorbidity index, one of the predictors of poor outcome (9, 13). The importance of comorbidities is underlined by a recent study which showed that comorbidities are the main determinants of prognosis in IE in elderly patients (10). Considering these data on the impact of cardiac predisposition, specifically a history of ICD implantation, poor dental status and the comorbidity CAD, our finding of their increased prevalence in men indicates an increased risk of developing IE and even a high risk of complications and poor outcome. Accordingly, assessment of these characteristics at the time of admission could facilitate the implementation of appropriate care strategies.

### Outcomes are worse in men

In line with their increased risk profile, we observed a more severe clinical course of disease in male patients. Our study found an increased prevalence of symptoms and increased severity of disease in men, as evidenced by higher rates of complications and adverse outcomes. Specifically, men had high and compared to women increased rates of heart failure with reduced ejection fraction and kidney failure, and spent a longer average time in the ICU. heart failure with reduced ejection fraction was observed in more than half of male patients, and renal failure requiring new-onset of dialysis was observed in almost a quarter of male patients. Finally, despite the risk factor of older age of women, men had a comparable absolute mortality rate and a higher mortality rate after adjustment for baseline characteristics. About 20% of male patients died during their initial hospital stay. In line with the results of this study, numerous previous studies have documented a high incidence and negative prognostic implications of organ failure in IE and a less favorable prognosis in men. A recent study also reported very high rates of serious cardiac and systemic complications of IE, with nearly 80% of patients experiencing at least one such complication (36). Similar to our findings, congestive heart failure has been identified as the most common cardiac complication, occurring in approximately 50%–60% of IE episodes (1). The implications thereof have been demonstrated since the complications of heart failure and valvular dysfunction are among the most important factors influencing clinical outcome. In particular, the International Collaboration on Endocarditis-Prospective Cohort Study showed that the presence of heart failure in IE patients was associated with a significantly higher in-hospital mortality of 29.7% compared with 13.1% in patients without heart failure (37). Consistent with our findings, acute kidney injury has also been described as a common complication of IE with recently reported rates of 39.8% and 52.7% (38, 39). Its prognostic impact has been outlined as the risk of death increases by 23.1% for every 10 ml/min decrease in estimated endogenous creatinine clearance using the Cockcroft-Gault formula (40). Consequently, the increased complication rates observed in men in our study suggest a higher risk of mortality. However, it is important to note that men are more likely to be younger (4), whereas older age is associated with increased mortality (6, 10, 14). While several studies report lower absolute mortality rates in men (5, 14), a US-wide analysis including patients aged 65 years and older found higher mortality rates in men (6). It is also noteworthy, that in the study cited above, which found higher mortality rates in women, the sex-difference was time-dependent. While women had increased 30-day and 1-year mortality, in-hospital mortality did not significantly differ to that of men (5). Consistent with this, women have been reported to have a longer recovery time (7). Our findings on higher complication rates and consistently elevated early mortality rate after adjustment for baseline characteristics in men during the initial hospitalization emphasize men's increased vulnerability in the early stages of IE.

### Early diagnosis is crucial in men

Given their more pronounced risk factors and increased early complication rates, it appears both feasible and necessary to reduce the time to diagnosis in men. The earlier onset of fatalities in men compared to women, combined with their more favorable long-term prognosis later on (5), underscores the potential of rapid diagnosis. In our study, a shorter time to diagnosis in men was associated with lower in-hospital mortality. In detail, men who were diagnosed within three and a half days had significantly lower in-hospital mortality rates, more than halved, compared to men with a longer delay in diagnosis. Notably, this finding was only seen in men, which may be due to a more rapid course of the disease in the early stage, as evidenced by their higher complication rates despite similar delays in diagnosis and initiation of antibiotic treatment. Consistent with our findings on the prognostic benefit of early diagnosis, it has been shown that patient outcomes in IE are highly variable and depend on prompt recognition and timely application of surgery when indicated (41), as targeted interventions are critical in reducing mortality (6). Thus, frequent delayed and missed diagnosis of IE affects patients' chances of recovery and survival (42). The recommendation to administer empirical antibiotics as soon as possible (43) and the reduction in mortality and embolic risk by early surgical intervention compared to medical therapy alone (44) highlight the critical role of prompt and targeted therapy. In line with our results, a recent study found a particularly pronounced benefit of early targeted therapy in the vulnerable elderly population, suggesting a particular benefit in high-risk patients (6). In consistence with this, our results show that early diagnosis is fundamental to improving outcomes in the particularly vulnerable male patient population. The more than 50% lower mortality rate in male patients diagnosed within 3.5 days impressively demonstrates the potential of early diagnosis in men to reduce the burden of IE.

### Male sex as a risk factor: implications for the diagnosis of IE

The result of our study, that in the majority of patients diagnosed with definitive IE, this diagnosis was made later during the hospital stay, but not at the time of admission, underlines the feasibility and need for enhancing the diagnostic process. The findings of sex-specific disparities, with a higher prevalence of predisposing conditions, a higher risk of complications descriptive to male sex, as well as the benefit of rapid diagnosis in this cohort, provide valuable approaches to improve the diagnostic algorithm. As the Duke criteria aim to reflect the risk of the presence of IE, with each criterion being associated with a certain risk increase, male sex qualifies as an additional criterion according to our findings. Consequently, adding male sex to these criteria can help to improve this diagnostic score, particularly in terms of sensitivity, which currently stands at only 84.2% (19). Furthermore, as sex is immediately and easily assessable, incorporation of this criterion can shorten diagnostic delay. Given the particular criticality of diagnostic delay in men, such a revision can provide a targeted improvement in this aspect as well. Additionally, the establishment of sex-specific recommendations for the time for completing diagnostic work-up or sex-specific modification of the recommended timing for repetition, i.e., of TTE/TOE (5–7 days) (10), can address this point and reflect the urgency of diagnosing IE in men. Thus, we argue for the inclusion of male sex in the diagnostic algorithm for IE.'

## Conclusion

This study evaluates sex-specific aspects in the management of IE that are not yet included in the guidelines. Considering our results, we can confirm the usefulness of the Duke criteria and other published risk factors for diagnosis and risk estimation in IE. As we found a higher prevalence of predisposing conditions and cardiac comorbidities in men, which may explain their worse, we would like to highlight the diagnostic potential of these conditions. In this course, we want to emphasize the criticality of diagnostic delay in men, particularly in light of the increased early complication rates and higher in-hospital mortality after adjustment for baseline characteristics. We therefore mitilate against a similar assessment of IE in men and women, as appreciation of the more pronounced risk profile associated with male sex could help to address the poorer prognosis of men. Moreover, we argue for considering the assessment of IE in men as an emergency.

## Limitations

The results of our study have to be interpreted with several confinements. As this is a retrospective, single-center study, it has several inherent limitations descriptive to this design. As the data collection was retrospective, some data were incomplete. As this was a single-center analysis, the number of patients included was relatively limited. Furthermore, the discrepancy in the number and the age of patients between the groups may be open to question. To address this issue, all patients in the defined time period were included without patient exclusion or pre-selection aiming to reduce selection bias as much as possible. Accordingly, these differences reflect the incidence and characteristics of the population, thereby providing an unbiased insight. However, we cannot exclude differences in certain baseline characteristics including socioeconomic state. Additionally, due to the explorative character of this study, all results must be interpreted as hypothesis-generating. Moreover, diagnostic assessment and treatment strategies may be a subject to debate due to their ongoing development. However, all diagnostic and therapeutic measures were indicated according to the then current guidelines and the Duke criteria remained stable over time (8, 9).

## Data Availability

The datasets presented in this article are not readily available because the medical data of the patients is restricted in access and secured with 2- factor authentication. Requests to access these datasets should be directed to lordayayi@yahoo.de.
