# Supplementary material for: Men's more frequent predisposing factors in infectious endocarditis facilitate improvement of outcomes by shortening of diagnostic delay
Source: Front Cardiovasc Med. 2025 Feb 12;11:1517288. doi: 10.3389/fcvm.2024.1517288 (PMC11860880; doi:10.3389/fcvm.2024.1517288)
Supplement: Supplementary file 1 [file Table1.docx]

| **Supplementary Table 1: Covariates in the multivariate analysis for in-hospital mortality including all baseline characteristics of significant difference in the univariate comparisons between the groups (p value < 0.05)** | | | |
| --- | --- | --- | --- |
|  | **p value** | **Hazard Ratio** | **95%-Confidence Interval** |
| Age | 0.892 | 1.004 | 0.954-1.056 |
| Known CAD | 0.192 | 0.396 | 0.099-1.591 |
| History of Myocardial Infarction | 0.324 | 1.746 | 0.577-5.290 |
| Predisposing Heart Disease or intravenous Drug Abuse | 0.244 | 2.007 | 0.621-6.488 |
| ICD | 0.203 | 0.407 | 0.102-1.623 |
| Poor Dental State | 0.962 | 0.000 | n.m. |
| Thyroid dysfunction | 0.145 | 0.143 | 0.010-1.960 |
| Splenomegaly | 0.833 | 0.882 | 0.277-2.815 |
| Location TV | 0.612 | 1.804 | 0.184-17.672 |
| Hypothyroidism | 0.175 | 5.381 | 0.472-61.280 |
| Predisposing Heart Disease | * |  |  |
| CAD, Coronary Artery Disease; ICD, Implantable Cardioverter-Defibrillator; TV, tricuspid valve;  n.m., not measurable  *not calculated because of constant or linearly dependent covariates | | | |
